# Human milk feeding and cognitive outcome in preterm infants: the role of infection and NEC reduction

**DOI:** 10.1038/s41390-021-01367-z

**Published:** 2021-06-24

**Authors:** Winok Lapidaire, Alan Lucas, Jonathan D. Clayden, Chris Clark, Mary S. Fewtrell

**Affiliations:** 1 UCL GOS Institute of Child Health, University College London, London, UK; 2 Department of Cardiovascular Medicine, University of Oxford, Oxford, UK

## Abstract

**Background:**

Breast milk has been associated with lower risk of infection and necrotising enterocolitis (NEC) and improved long-term cognitive outcomes in preterm infants but, if unsupplemented, does not meet the nutritional requirements of preterm infants.

**Methods:**

Preterm infants were randomised to receive a high nutrient intervention diet: preterm formula (PTF) or the standard diet: term formula (TF) or banked donor breast milk (BBM), either as their sole diet or as supplement to maternal breast milk (MBM). IQ tests were performed at ages 7, 15, 20, and 30 years.

**Results:**

An increase in MBM and BBM intake was associated with a lower chance of neonatal infection/NEC. Neonatal infection/NEC was associated with lower Full Scale IQ (FSIQ) and Performance IQ (PIQ) score at ages 7 and 30 years. The relationship between higher intake of MBM and PIQ at age 7 years was partly mediated by neonatal infection/NEC. The intervention diet was associated with higher Verbal IQ (VIQ) scores compared to the standard diet. There was no evidence that these effects changed from childhood through to adulthood.

**Conclusions:**

Neonatal diet is an important modifiable factor that can affect long-term cognitive outcome through a ‘human milk’ factor, protecting against infection/NEC, and a ‘nutrient content’ factor.

**Impact:**

- This is the first study to demonstrate the effects of neonatal infection/necrotising enterocolitis (NEC) on IQ in the same cohort in childhood and adulthood.
- Diet can be a key factor in long-term cognitive outcome in people born preterm by preventing neonatal infection/NEC and providing adequate nutrients.
- Human milk, whether MBM or BBM, is associated with a reduced risk of infection/NEC.
- A higher nutrient diet is associated with better cognitive outcome in childhood.
- Performance IQ is particularly vulnerable to the effects of infection/NEC and verbal IQ to the quantity of (macro)nutrients in the diet.

## Introduction

Infection and necrotising enterocolitis (NEC) are common among preterm infants during their hospital stay.^1^ In addition to significant short-term morbidity and mortality, the systemic inflammatory response to infection and NEC can be harmful for the developing brain of the preterm infant.^2^ Inflammation activates microglia, which releases free radicals that damage pre-oligodendrocytes during the preterm period, disturbing white matter development and causing white matter injury.^3^ This has been associated with long-term changes in brain micro-structure^4,5^ and lower intelligence quotient (IQ).^5^

Neonatal diet plays an important role in preventing infection and NEC in preterm infants. Intake of breast milk during the first days of life has been shown to be associated with decreased infection, morbidity, and mortality in very low birth weight (BW) infants.^6–8^

Preterm infants exclusively fed human milk were up to 6–10 times less likely to suffer from NEC than infants fed cow milk-based formula,^8,9^ with a strong dose–response relationship.^10^ This could be due to beneficial effects of specific human milk components on the infant immune system or to avoidance of deleterious effects related to exposure to cow’s milk.^11,12^ However, providing breast milk can be challenging for mothers after preterm delivery, so many preterm infants receive either banked donor breast milk (BBM) or preterm formula (PTF) if maternal breast milk (MBM) is unavailable or insufficient to meet requirements. Pasteurisation and freezing of BBM may reduce levels of nutrients and anti-infective agents, thus equivalence with MBM cannot be assumed.^13,14^ Nevertheless, Lucas et al. showed that the type of breast milk did not affect the incidence of NEC.^9^

Current evidence suggests that both intake of human milk and adequate nutrient intake favourably influence cognitive outcome in preterm infants, possibly by different mechanisms.^15^ Despite the low nutrient content of the BBM used in the trial by Lucas and colleagues, infants receiving this diet scored equally well on Bayley developmental indices at 18 months of age compared to those who received nutrient-enriched PTF and significantly higher than those fed standard term formula (TF) with low nutrient content.^16^ The positive effect of human milk on cognitive outcome could potentially partly be explained by its protective effect against infection or its moderating effect on inflammation. However, no study has yet investigated associations between early diet, infection, and long-term cognitive outcome.

We hypothesise that (i) higher human milk intake (MBM or BBM) is associated with lower rates of neonatal infection/NEC and (ii) absence of infection/NEC is associated with higher IQ outcomes. We also hypothesise that (iii) there is a direct relationship between early diet and IQ outcomes and (iv) the relationship between human milk and IQ can (in part) be explained by effects on the occurrence of infection or by modifying the harmful impact of infection/NEC on IQ. Using data from a large prospective diet intervention trial with four follow-up time points allows us to see if any effect of diet and infection/NEC on IQ remained over time.

## Methods

### Original study design

**Fig. 1:**
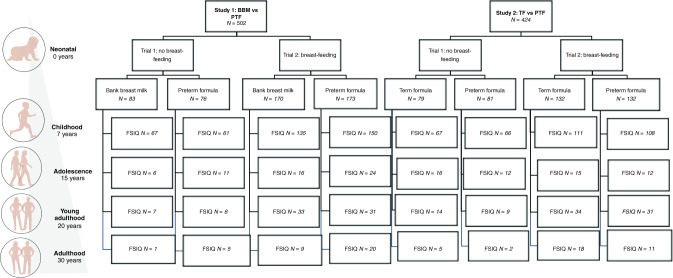
Study design flowchart. Original study design with subject numbers at different follow-up stages.

Between 1982 and 1985, 926 infants with BWs <1850 g were recruited for a diet intervention trial from 5 neonatal units in England (study 1: Cambridge, Ipswich, and King’s Lynn; study 2: Norwich and Sheffield). The study design is shown in Fig. 1 and macronutrient content of diets in Table 1. The original trial had a randomised control design, where all participating newborns were allocated to receive an intervention diet (PTF) or the standard diet (BBM or TF). Depending on the mother’s choice to provide breast milk, infants either received the randomised diet as their sole source of nutrition (trial 1) or as a supplement to mother’s expressed breast milk (MBM; trial 2). All infants thus received 100% MBM, BBM, TF, or PTF or a combination of MBM with BBM, TF, or PTF. Since the proportion of MBM intake varied considerably between infants, a proportional intake for each diet was calculated.

**Table 1:** Macronutrient content of trial diets.

	PTF	TF	BBM	MBM
Energy (kcal/dl)	80	68	46	62
Protein (g/dl)	2.0	1.5	1.1	1.5
Fat (g/dl)	4.9	3.8	2.0	3.0

Human milk values are based on samples from pooled 24-h milk collections.

In addition to containing more protein and fat than TF (see Table 1), PTF was also enriched in sodium, calcium, phosphorus, copper, zinc, vitamins D, E, and K, water-soluble vitamins, carnitine, and taurine (Farley Health Products). BBM was drip milk from mothers with term born infants, which was pasteurised and frozen. The expressed MBM was not pasteurised and only occasionally frozen. Milk fortifiers were not available at the time and nutrient supplements were not used.^17^ In infants fed BBM or MBM, a sample was collected from each pooled 24-h milk collection. The nutritional values in Table 1 are based on averages of these samples.^17,18^

Enteral feeds, given by nasogastric tube, were increased, according to tolerance, with a target intake of 180 ml/kg/day. Forty-three percent of infants required initial parenteral nutrition, including amino acid infusion (with or without lipid). There was no difference in days until full enteral feeds between randomised feed groups.^19^ Enteral intake was recorded daily as part of the study and episodes of NEC and infection were recorded prospectively by research staff during the hospital stay.

Participation in the trial ended at discharge or when the infant reached 2000 g, whichever occurred first. Researchers and nurses were blinded to group membership when conducting follow-ups. Intake of diets is expressed as percentage of total enteral intake. NEC was classified using the British Association for Perinatal Pediatrics classification.^20^ Grade 1 cases had at least two of the following NEC features: (i) pneumatosis intestinalis on abdominal radiograph, (ii) abdominal distention and/or free air in the abdomen or frothy appearance of bowel lumen as seen on a radiograph, (iii) blood in the stool, (iv) lethargy, hypotonia, and/or apnoeic episodes. Grade 2 cases showed at least one of the following additional more serious symptoms: (﻿abdominal tenderness or rigidity, tissue in stool, abnormal bleeding with trauma, spontaneous bleeding, peripheral white blood cell count <6 × 10^9^/l or peripheral platelet count <100 × 10^9^/l at the time of illness, gas in the portal vein, or free air in the abdomen visible on an abdominal radiograph).^9^ The cases were further divided into confirmed cases, which were diagnosed based on radiological features or during surgery, and unconfirmed cases. This study classified confirmed and unconfirmed grade 1 and grade 2 cases as NEC. Infection was classified by a positive blood culture and/or increased white blood cell count.

### Follow-up studies

All children who could be contacted and consented were followed up at age 7 years and assessed with the abbreviated version of the Wechsler Intelligence Scale for Children WISC-R. At age 15 years, only participants born ≤30 weeks gestational age (GA) who had been judged neurologically normal at 7 years were invited to take part. The full versions of the Wechsler Intelligence Scale for Children—Third edition (WISC-III) or Wechsler Adult Intelligence Scale—Revised (WAIS-III) were administered. At 20- and 30-year follow-up studies, all surviving members who were seen at age 7 years who could be contacted and were willing to take part completed the WAIS.

### Statistical analyses

Statistical analyses were performed using the R software.^21^ Each analysis was adjusted for maternal education, BW, GA, sex, and covariates related to illness; the number of days of ventilation, and the number of days until 150 ml/kg/day of enteral feeds was reached. Adjustment for multiple comparisons across variables was done with the Benjamini and Hochberg method.^22^ Longitudinal mixed models were used to examine the effects of diet and infection on IQ scores across time points.

A logistic regression was used to examine whether diet predicted occurrence of infection/NEC. analysis of covariance models were used to examine infection/NEC and diet variables as predictors of Full Scale IQ (FSIQ), Verbal IQ (VIQ), and Performance IQ (PIQ) scores, using IQ scores at ages 7, 15, 20, and 30 years. An interaction term between the infection and diet variables was added to determine whether there was a moderating effect of diet on the relationship between infection and IQ.

For the analyses where diet and infection were significant predictors of the same IQ outcome, a mediation analysis was performed. A mediation analysis tests whether a relationship (in this case between diet and IQ outcomes) weakens when another factor (the mediator: infection) is included in the regression.^23^ If this is the case, there is a relationship between the predictor and the mediator and the mediator and the outcome that explains part of the common variance between the predictor and the outcome. In other words, the relationship between the predictor and the outcome is (partially) mediated by the mediator.

A mixed model was used to investigate whether effects of neonatal infection and early diet on IQ scores changed over time using the lme function from the lmer package in R. Subjects included in the analysis were all part of the 7-year follow-up, but not all subjects had IQ scores at subsequent time points. List-wise deletion was applied, so that even subjects with no IQ scores at ages 15, 20, or 30 years could be included in the analysis. These data points were approximated using iterative procedures to get the maximum likelihood estimates. In addition to the principal analyses, interaction terms were added to test whether the effect of infection and diet on IQ outcome was different for boys and girls.

## Results

### Population characteristics

Table 2 describes the characteristics of participants included at birth and at each follow-up. The mean proportional intake of diets, percentage of subjects in the low nutrient group, and percentage of subjects with neonatal infection/NEC did not differ significantly between the original cohort and the participants in the different follow-up studies. Since only participants born ≤30 weeks GA who had been judged neurologically normal at 7 years were invited to take part in the study at age 15 years, the GA and BW was lower in this sample by design. This was also reflected in illness severity factors such as mean days of ventilation and mean days to achieve 150 ml/kg/day of enteral feeds. At age 20 years, these factors were again similar to the original cohort averages. Subjects who participated at ages 15 and 20 years were more likely to be female and have a mother with higher educational attainment than the subjects who did not participate.

**Table 2:** Population characteristics.

	At birth	Age 7 years	Age 15 years	Age 20 years	Age 30 years
Number of subjects included in the study	926	765	112	167	71
Mean (SD) GA in weeks	31 (2.8)	31 (2.7)	29*** (1.9)	31 (2.6)	30 (2.4)
Mean (SD) BW in grams	1369 (316)	1402 (294)	1208*** (275)	1390 (299)	1308 (300)
Percentage of male subjects	51	50	51	35	46
Percentage of subjects with maternal education less than A-levels	51	51	41*	36*	34*
Mean (SD) days of ventilation	5 (9.8)	3 (6.9)	6 (8.3)	3 (6.2)	5 (9.0)
Mean (SD) days until 150 ml/kg/day of enteral feeds	12 (9.0)	11 (8.3)	15 (10.6)	11 (7.2)	12 (9.0)
Mean (SD) days in trial	33	36 (21)	45 (26)	36 (17)	44 (23)
Mean % MBM	32 (37)	32 (37)	36 (41)	41 (38)***	45 (39)***
Mean % BBM	19 (35)	17 (34)	11 (27)***	12 (29)**	7 (21)***
Mean % PTF	34 (42)	34 (42)	34 (42)	27 (37)	28 (37)
Mean % TF	15 (33)	16 (34)	19 (37)	20 (36)	20 (36)
% in the standard diet group	50%	50%	47%	53%	46%
% subjects with infection/NEC	14%	12%	17%	11%	15%
FSIQ score, mean (SD)	–	100.5 (16.7)	96.1 (14.8)	103.6 (17.2)	107.1 (15.0)
VIQ score, mean (SD)	–	99.7 (18.8)	97.1 (14.7)	101.1 (20.0)	106.0 (15.7)
PIQ score, mean (SD)	–	100.8 (16.5)	96.2 (15.0)	104.8 (14.5)	106.5 (14.4)

Significant differences between cohort at birth and follow-up samples: *p < 0.05, **p < 0.01, ***p < 0.001 as measured by *t* tests or chi-square tests.

### Diet and infection/NEC

Each 10% increase in MBM and BBM intake was associated with approximately a 8 and 12% lower chance of neonatal infection/NEC, respectively. A 10% increase in TF was associated with a 12% increase in the chance of infection/NEC. There was no difference in odds of developing infection/NEC between the standard and intervention diet groups (Table 3).

**Table 3:** Relationship between nutrition and infection occurrence using a logistic regression model adjusted for maternal education, sex, birth weight, gestational age, days of ventilation, and the number of days until 150 ml/kg/day of enteral feeds (*n* = 765).

Nutrition type	Infection	
	Change in odds per % increase diet intake	95% CI lower–upper
% MBM	0.992*	0.984–1.000
% BBM	0.988**	0.976–0.997
% TF	1.012***	1.004–1.019
% PTF	1.003	0.997–1.010

	Odds ratio	95% CI lower–upper
Standard (TF/BBM) vs intervention (PTF) diet group	0.657	0.372–1.145

Values in italics are significant after adjusting for multiple comparisons. The odds ratio changes are for each percentage increase in each of the diets or for allocation to the standard vs intervention group.

*p < 0.05, **p < 0.01, ***p < 0.001.

### Infection/NEC and IQ at ages 7, 20, and 30 years

**Fig. 2:**
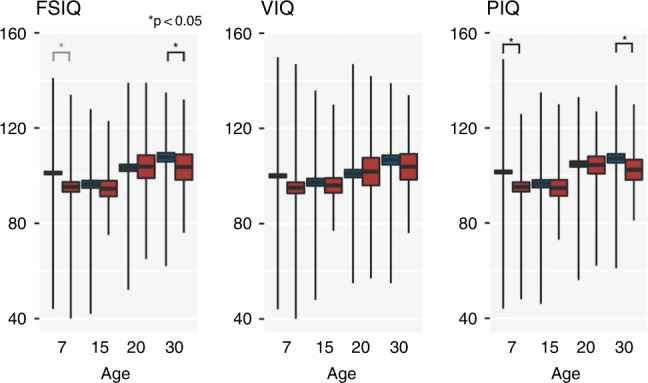
IQ scores at ages 7, 15, 20, and 30 years of participants with (red) and without (blue) neonatal infection/NEC *p < 0.05. The box around the mean shows the standard error range, and the vertical lines show the range of values (minimum and maximum values).

At both ages 7 and 30 years, neonatal infection/NEC was associated with a lower FSIQ and PIQ score. The relationship between infection/NEC and FSIQ at age 7 years did not remain significant after multiple comparison corrections. Although the mean IQ scores were consistently lower in the infection/NEC group, there were no significant relationships between infection/NEC and IQ at ages 15 and 20 years (see Fig. 2 and Table 4).

**Table 4:** Relationship between infection and IQ outcomes at ages 7, 15, 20, and 30 years using an ANCOVA model adjusted for maternal education, sex, birth weight, and gestational age, days of ventilation, and the number of days until 150 ml/kg/day of enteral feeds (age 7 years: FSIQ *n* = 660, VIQ *n* = 669, PIQ *n* = 662, age 15 years: FSIQ *n* = 99, VIQ = 100, PIQ = 100, age 20 years: FSIQ *n* = 145, VIQ = 145, PIQ = 145, age 30 years: FSIQ = 63, VIQ = 64, PIQ = 63).

Age (years)	FSIQ score			VIQ score			PIQ score		
	No infection/NEC, mean (SD)	Infection/NEC, mean (SD)	*F*-statistic	No infection/NEC, mean (SD)	Infection/NEC, mean (SD)	*F*	No infection/NEC, mean (SD)	Infection/NEC, mean (SD)	*F*-statistic
7	101.1 (15)	95.3 (19.6)	4.2*	100.1 (18.2)	95.1 (22.6)	0.4	101.5 (16.0)	95.3 (18.9)	6.0*
15	96.5 (14.8)	94.6 (14.9)	0.0	97.5 (14.8)	96.1 (14.3)	1.0	96.6 (14.9)	94.8 (15.6)	0.1
20	103.3 (17.0)	103.8 (20.3)	0.5	101.1 (19.5)	101.9 (24.5)	0.7	104.8 (14.5)	104.4 (15.4)	1.5
30	107.8 (14.5)	103.6 (17.7)	5.8*	106.8 (15.4)	103.9 (17.8)	0.1	107.2 (14.4)	102.5 (14.0)	5.3*

Values in italics are significant after adjusting for multiple comparisons across IQ variables for each time point. The *F*-statistic is the mean square of the independent variable divided by the mean square of the residuals. A larger *F*-statistic indicates a higher likelihood that the variation caused by the independent variable is real and not due to chance.

*p < 0.05.

### Diet and IQ at ages 7, 15, 20, and 30 years

Each 10% higher MBM intake was associated with 0.7 higher FSIQ, 0.8 higher VIQ, and 0.6 higher PIQ scores at age 7 years. It was also associated with higher IQ scores at age 15 years, but these results did not survive multiple comparisons corrections. In contrast, each 10% increase in BBM intake was associated with 0.6 lower FSIQ and 0.9 lower VIQ scores at age 7 years. The negative association between BBM and VIQ at age 20 years did not survive multiple comparison corrections. The intervention diet (PTF) was associated with 3.2 higher VIQ scores at age 7 years compared to the standard diet (BBM/TF; see Table 5).

**Table 5:** Relationship between diet and IQ outcomes at age 7 years using a general linear model adjusted for maternal education, sex, birth weight, gestational age, days of ventilation, and the number of days until 150 ml/kg/day of enteral feeds (age 7 years: FSIQ *n* = 660, VIQ *n* = 669, PIQ *n* = 662, age 15 years: FSIQ = 99, VIQ = 100, PIQ = 100, age 20 years: FSIQ *n* = 145, PIQ = 145, VIQ = 145, age 30 years: FSIQ = 63, VIQ = 64, PIQ = 63).

Nutrition type	Age	FSIQ score			VIQ score			PIQ score		
		Estimate	95% CI		Estimate	95% CI		Estimate	95% CI	
% MBM	7	0.07***	0.04	0.10	0.08***	0.04	0.11	0.06***	0.02	0.09
	15	0.09*	0.02	0.16	0.08*	0.01	0.15	0.08*	0.01	0.15
	20	0.06	−0.02	0.13	0.05	−0.03	0.13	0.05	−0.01	0.12
	30	0.04	−0.05	0.13	0.01	−0.09	0.12	0.06	−0.04	0.15
% BBM	7	−0.06***	−0.09	−0.03	−0.09***	−0.13	−0.05	−0.03	−0.06	0.01
	15	−0.04	−0.15	0.06	−0.04	−0.14	0.06	−0.03	−0.14	0.18
	20	−0.07	−0.16	0.02	−0.04	−0.13	0.06	−0.09*	−0.17	−0.02
	30	−0.02	−0.19	0.14	−0.07	−0.24	0.11	0.02	−0.14	0.18
Intervention vs standard diet group	7	1.79	−0.37	3.95	3.21*	0.64	5.79	−0.01	−2.38	2.37
	15	2.99	−2.53	8.51	4.61	−0.65	9.88	−0.11	−6.03	5.81
	20	1.74	−3.45	6.92	0.99	−4.83	6.82	2.18	−2.47	6.84
	30	2.27	−4.63	9.17	4.31	−3.28	11.91	−0.88	−7.79	6.04

Values in italics are significant after adjusting for multiple comparisons across IQ and nutrition variables for each time point. The estimate indicates the change in IQ points per 1% increase in BBM/MBM in the diet or difference in IQ points between the intervention group and the standard diet group (positive values indicate a higher IQ in the intervention group).

*p < 0.05, ***p < 0.001.

### Mediation/moderation effect of infection/NEC on the diet–IQ relationship

Only MBM and infection/NEC were both associated with PIQ at age 7 years and with each other. The relationship between MBM and PIQ was significantly mediated by reduced infection/NEC (5% mediated, *p* < 0.05).

There was no interaction effect between diet and infection/NEC with regard to IQ outcomes. In subjects who developed infection/NEC, diet was not related to FSIQ or PIQ, but BBM intake was associated with lower VIQ (estimate: −0.23, *p* < 0.05, confidence interval (CI) [−0.42, −0.03]).

### Effects of time and sex

The main effects of diet and infection/NEC on IQ scores were very similar using a mixed model including IQ scores at ages 15, 20, and 30 years. Neonatal infection/NEC was associated with lower FSIQ (estimate −4.18, 95% CI [−8.21, −0.15], *p* = 0.04) and PIQ (estimate −5.09, 95% CI [−9.32, −0.85], *p* = 0.01), whereas the standard diet was associated with lower VIQ scores relative to the intervention diet (estimate 3.20, 95% CI [0.70, 5.70], *p* = 0.01). Adjusting for confounding factors, there was no significant interaction effect between time and diet or infection/NEC on IQ scores. There was no significant effect of sex on the relationship between diet and the odds of infection or on the relationship between infection/NEC and IQ outcomes.

## Discussion

### Diet and infection/NEC

Increased human milk intake, whether MBM or BBM, was associated with reduced risk of infection/NEC. This study is in accordance with other studies reporting associations between an exclusively human milk-based diet and lower NEC rates.^24,25^ Systematic reviews suggest that the use of BBM has a protective effect against NEC in preterm infants.^26^ Because diet intakes are relative and always add up to 100% in each individual, a lower intake of human milk automatically means a higher intake of formula and vice versa. It is therefore difficult to establish whether human milk is actively protective against infection/NEC and/or formula is causing infection/NEC.

### Early life factors and IQ outcomes

Neonatal infection/NEC was associated with lower FSIQ and PIQ scores at age 7 years. Additionally, the current results showed a positive association between MBM intake and all IQ scores. This is consistent with a previous publication from this cohort at age 15 years reporting a significant positive relationship between VIQ and intake of mother’s milk^27^ as well as another recent study.^28^ However, despite its protective effect against infection, BBM intake was associated with lower FSIQ and VIQ scores at age 7 years. This may be explained by the extremely low energy and protein content of the unfortified drip milk that was used at the time of the trial. The low-nutrient diet category, a combination of the BBM and TF groups, showed a similar association. This is consistent with the negative effect of the standard diet that was relatively low in nutrients on IQ scores reported previously in this cohort. Boys fed on TF as their sole diet had a significantly lower VIQ score at age 7 years compared to boys fed PTF^29^ and infants fed BBM or TF had significantly lower VIQ at age 15 years.^30^ The nutrient content of current BBM is much higher because drip milk, which has lower fat and energy content than expressed milk, is no longer used and BBM is routinely fortified. Therefore, this negative association with VIQ scores may no longer exist in modern day situations.

### Mediation/moderation

**Fig. 3:**
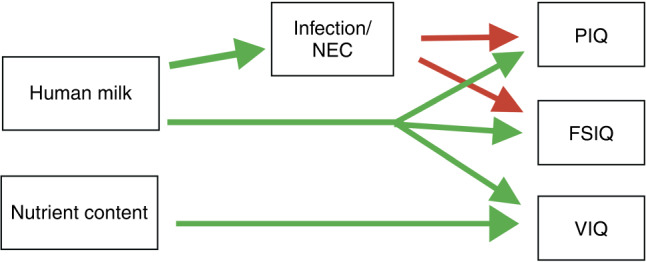
Suggested relationship between nutrition, infection, and IQ outcomes. Positive effects (reduced infection/NEC and increased IQ scores) are shown in green, negative effects (decreased IQ scores) are shown in red.

The positive relationship between MBM and PIQ scores was partly mediated by a reduction in infection/NEC, whereas the relationship between MBM and VIQ and FSIQ was independent of neonatal infection/NEC (Fig. 3). These results suggest that MBM may improve cognitive outcome partly by reducing the risk of infection/NEC. The effect was small, which could in part be due to the relatively small proportion of subjects with infection/NEC, yet significant. We did not find any evidence for a moderating effect of diet on the negative association between infection/NEC and subsequent IQ scores.

**Fig. 4:**
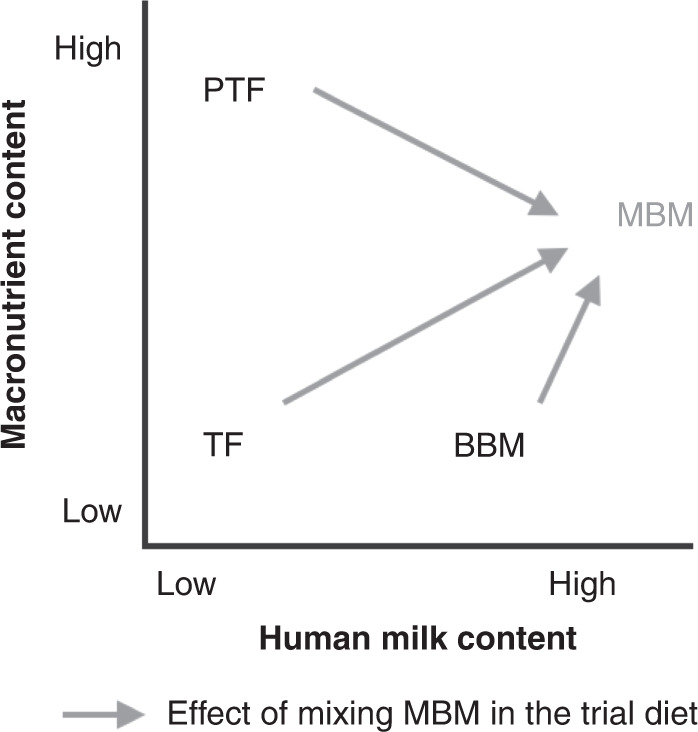
Schematic representation of the human milk and macronutrient content level in each of the diets. TF has low macronutrient and low human milk content, PTF has the high macronutrient content and low human milk content, BBM has low macronutrient and medium-high human milk, and MBM has medium macronutrient and high human milk content.

Our results suggest that there are two important factors in neonatal diet: a human milk factor and a nutrient content factor (Fig. 4). We discerned a pathway linking human milk intake to reduced infection/NEC and absence of infection/NEC with higher PIQ scores, while the high nutrient intervention diet^31^ is associated with higher VIQ scores unrelated to infection/NEC.

BBM does have the positive human milk factor, which is reflected in association with reduced infection/NEC and higher FSIQ scores. At the same time, it lacks adequate nutrient content, explaining the negative relationship with VIQ scores. Using fortified BBM could theoretically retain the protective effects of human milk against infection/NEC while providing the infant with sufficient nutrients.

### Time and sex effects

There was no evidence that the association between infection/NEC and lower IQ outcomes changed over time. The relationship between the proportion of intake of diets and infection/NEC and IQ was also not significantly different between boys and girls. Of note, this seems to be in contrast with previous studies in this cohort that reported a greater direct relationship between diet and IQ in boys compared to girls. For example, the beneficial effect of PTF compared to TF on VIQ and FSIQ scores at age 7 years was significant in boys but not in girls,^29^ while MBM intake was significantly correlated with all IQ scores at age 15 years in boys and only with VIQ in girls.^27^ It is possible that it was no longer possible to detect the difference in relationship between diet and IQ between male and female groups due to selective attrition of boys with poorer outcomes, which may have artificially reduced the difference in IQ between the male and female groups at later follow-up studies. Indeed, the difference between male/female IQ points at age 7 years is 1.8 IQ points in the group who came back for the 30-year follow-up, compared to 2.1 in the total group.

### Strengths and limitations

An important strength of the study is that collection of data on nutritional intake and infection/NEC was recorded prospectively by dedicated research nurses. Second, this trial was conducted before fortifiers were routinely added to human milk and therefore, unlike many current day trials in which human milk is mixed with cow’s milk-based fortifiers, it is possible to examine the relationship between cow’s milk and human milk and infection/NEC. Being the first randomised diet study in preterm infants, this study presents a unique opportunity to study long-term effects of neonatal diet. Since current day feeding practices have improved since this trial was conducted, there is a larger variation in nutritional content in this study than we currently see in practice. Nevertheless, the findings are still relevant as modern preterm infants also have on average a lower GA and BW, are sicker, and still often receive insufficient nutrients. Furthermore, the large nutritional variation provides a better opportunity to detect relationships between nutrient content of neonatal diet and outcome. Conversely, if no significant relationship can be found between infants who experienced large differences in neonatal diet in this study, it is perhaps less likely that smaller modifications to the already much improved modern day neonatal diet will have a significant effect on the long-term outcome.

Combining infection and NEC into one category increased the power of this study but might mask some specific effects of either illness. Furthermore, as previously reported in more detail by Lucas and colleagues, there were subclassifications of NEC that indicated severity and certainty of diagnosis.^9^ In this study, classification of NEC did not require confirmation of diagnosis by surgery or at necropsy nor any of the grade 2 NEC features.^20^ Furthermore, infection classification required only a positive blood culture or an increased white blood cell count. Blood cell count is a rapid, inexpensive, and widely available test to evaluate the likelihood of sepsis in a neonate with signs of infection, but it has limited sensitivity to infection.^32^ Nevertheless, an elevation in white blood cells is an indicator of a systemic inflammatory response that could potentially be harmful for the developing brain of the preterm infant. In future study designs, levels of pro-inflammatory cytokine profiles would provide a more precise and sensitive marker of potentially harmful inflammation.^33^ At the time this trial was conducted, such tests were not yet available.^34^ The study had a large sample size and data on diet and infection/NEC was collected prospectively and in a standardised manner. At age 7 years, 765 of subjects who survived were followed up. However, the sample sizes of the 15-, 20-, and 30-year follow-ups were much smaller. Maternal education of these subjects was on average higher than the original birth cohort average, and due to selection criteria, the sample at age 15 years had a lower GA and BW. The non-random missingness may have affected the validity of the mixed model analysis, because it reduces the accuracy of the maximum likelihood estimates of the missing data. This only had a limited effect on the overall longitudinal estimate, since this bias only affects one out of four time points on a subset of subjects. It should be kept in mind when reviewing the 15-year cross-sectional analyses that these were done on a sample of subjects <30 weeks GA and results may therefore not automatically generalise to the complete sample.

Maternal education, maternal age, birth rank, marital status, the infant’s sex, and the model of delivery had a significant effect on the choice to express breast milk in this trial.^35^ Since it is unethical to prevent mothers from providing breast milk for their child, this is an important confounding factor in research on the effects of breast milk. All analyses were adjusted for maternal education, but the possibility of residual confounding remains. Factors such as interaction with parents and exposure to ongoing social adversities were not recorded and could therefore not be taken into account. It cannot be excluded that confounding factors that were not accounted for affected risk of NEC/infection, intake of MBM, and IQ could have been the primary driver of the described relationships. For example, if maternal illness can affect ability for the mother to produce milk and infant vulnerability to disease.

### Future studies

To further elucidate the apparent dissociation between human milk and infection/NEC compared to the amount of nutrients in the diet in terms of cognitive domains, future studies should investigate these relationships using more specific psychometric tests. The use of brain magnetic resonance imaging scans could further elucidate the mechanisms behind the different effects of human milk intake and the amount of nutrients in the diet on VIQ and PIQ. It would be particularly interesting to see if there is a difference in white matter microstructure between subjects with and without neonatal infection and of the language networks between subjects in the standard and intervention diet groups. In addition, for clinical purposes studies should investigate whether fortifying BBM could improve outcome in preterm infants by retaining the protective effect against infection/NEC and providing a high amount of nutrients.

## Conclusion

This study suggests that human milk, whether MBM or BBM, protects preterm infants against infection/NEC, and the absence of infection/NEC is associated with better FSIQ and PIQ outcomes. Protection against neonatal infection/NEC may thus be an important mechanism explaining the beneficial effect of human milk on cognitive outcome. Adequate nutritional content remains important, as shown by the lower VIQ scores in the low-nutrient diet group, but this was unrelated to infection/NEC.
